# Contextual influences of illicit adolescent marijuana cultivation and trading in the Inqguza Hill local municipality of South Africa: implications for public health policy

**DOI:** 10.1186/s13011-020-00338-7

**Published:** 2021-01-07

**Authors:** Emmanuel Manu, Mbuyiselo Douglas, Mohlomi Jafta Ntsaba

**Affiliations:** 1 Department of Population and Behavioural Sciences, School of Public Health, University of Health and Allied Sciences, Hohoe, Ghana; 2 Department of Nursing and Public Health, University of Kwazulu Natal, Durban, South Africa; 3 Department of Nursing, Walter Sisulu University, Nelson Mandela Drive, Mthatha, South Africa

**Keywords:** Adolescent, Eastern Cape Province, Marijuana cultivation, Inqguza Hill local Municipality, Socio-ecological theory, South Africa

## Abstract

**Background:**

Although commercial cultivation and trading of marijuana, commonly known as cannabis or dagga in the South African context, remains an illicit practice, adolescents actively engage in it. However, contextual influences that sustain adolescent involvement in illicit marijuana-related activties remain empirically unascertained.

**Objective:**

This study sought to ascertain the various contextual influences of adolescent illicit marijuana cultivation and trading in two communities in the Ingquza Hill Local Municipality (IHLM) of South Africa, using the tenets of the Socio-Ecological Model (SEM).

**Methods:**

The study utilised focus group discussions approach to interview thirty-three purposefully sampled participants who were recruited through the snowball sampling technique. A semi-structured interview guide was used to conduct the interviews, while thematic content analysis was used to analyse the data.

**Results:**

We found that illicit adolescent marijuana cultivation and trading was influenced by eleven contextual factors that are grouped under four levels of socio-ecological influence. These include intrapersonal influences (knowledge and skills in marijuana cultivation and courage), interpersonal influences (peer and family influences), communal level influences (economic reasons, early childhood exposure to marijuana activities, protection of family lands, the topography of the area and soil fertility) and policy-related influences (lack of communal bylaws on marijuana activities and laxity in law enforcement).

**Conclusion:**

It is recommended that substance abuse prevention policies and programmes focus on discouraging children from engaging in illicit marijuana activities in IHLM across the four tenets of SEM and curtailing adolescent involvement in marijuana cultivation and trading. There is also the need to incorporate the law enforcement approach into demand reduction strategies of the National Drug Master Plan (NDMP), which employs only an educative approach in its current form. Working agreements between municipal authorities, law enforcement agents and social service professionals also need to be strengthened to push demand reduction strategies for marijuana in communities to protect the rights of children as enshrined in the Children’s Act, 38 of 2005.

## Background

Marijuana, commonly known as dagga or cannabis in the South African context [1], is a gateway drug to more challenging illicit drug use such as cocaine. Hence, the involvement of adolescents in unlawful marijuana-related activities, such as its cultivation and trading, is of public health concern. Although the plant is commonly referred to as dagga in the South Africa context, the term “marijuana” is extensively used in the literature, making it an acceptable name for the plant worldwide [2]. The involvement of adolescents in drug-related activities such as the illicit cultivation and trading of dagga is not unique to South Africa. Studies have shown that a significant number of adolescents engage in illegal cultivation and trafficking of drugs in the Asia-Pacific region, particularly in Indonesia, the Philippines and Thailand [3, 4]. Adolescents’ involvement in illicit drug production and trading not only exposes and initiates them into the world of illegality, criminality, and hard drug usage, but also makes them vulnerable to harassment and exploitation by drug dealers and law enforcement agents [5]. They also face the risk of arrest and prosecution [6, 7].

Illegal marijuana cultivation and trading in South Africa is a significant challenge to the government [8]. Even though commercial marijuana cultivation and trading is prohibited [9], the provinces of Kwazulu-Natal (KZN) and the Eastern Cape (EC) have been noted as principal marijuana growing areas in the country [8]. In the Eastern Cape Province, the plant is extensively cultivated in communities along the coastal belt of the former Pondoland region where Ingquza Hill Local Municipality (IHLM) is located [10]. The cultivation and trading of marijuana in the area have been blamed on the lack of employment opportunities. While those employed receive salaries and are well off, most rural dwellers live in abject poverty, with their limited income from social security grants or remittances from family members living outside the region or in cities within the area [11]. As families try to find alternative income sources, some turn to illicit marijuana cultivation and trading, involving children and adolescents in the process [10, 12]. Land tenure security has also been cited as one of the reasons for illicit drug cultivation. Families struggle to maintain ownership of their lands in illegal drug production environments, hence, continuous land usage through illicit drug cultivation becomes a viable alternative [13].

Meanwhile, marijuana cultivation in South Africa has a long history that dates back to the fifteenth century AD. Asian merchants introduced the plant to the eastern coast of Africa in the thirteenth century. The plant then spread to Southern Africa by the fifteenth century [14]. The proliferation of marijuana cultivation and usage in South Africa, after its introduction into the country, led to the need for legislation to deal with marijuana cultivation, trading and use, which was first introduced in 1928. The legislation was backed by strict control of marijuana activities by tribal elders at the time whose power and control over the youth have waned over time, allowing children to indulge in illicit marijuana activities [14].

Several factors have been cited for adolescent involvement in illicit drug activities, such as marijuana cultivation and trading. For instance, poverty is one of the main factors that contribute to illegal adolescent drug production and trading [15, 16]. This is the case for IHLM in South Africa, where marijuana is cultivated and traded illegally [10]. Beyond economic reasons, societal influences have been identified as playing a key role in promoting illicit drug production or cultivation and trading [17, 18]. However, in the South African context, empirical evidence of factors that influence illicit drug production, especially among adolescents in poor communities, such as IHLM, except for economic reasons [10], is limited.

Moreover, studies on the adolescent-illicit drug nexus in the country mainly focus on knowledge and perceptions of illicit drug trafficking, prevalence of use, drug users’ experiences and interventions to curb adolescent substance use [19–23]. Also, there is paucity of data on policy implications of adolescent marijuana cultivation and trading in South Africa in general and vulnerable ecological settings of the country in particular. Therefore, we contextually explored the influences of illicit adolescent marijuana cultivation and trading in IHLM, which is known to be socio-economically marginalised [24], to inform policy on illegal adolescent marijuana cultivation and trading control in the municipality. Findings of the study would also be of interest to various professionals such as psychiatrists and social workers who bear the burden of rehabilitating marijuana and other substance abusers in the country. The study also adds to the body of knowledge on illicit adolescent marijuana-related activities on the African continent.

## Theoretical framework

**Fig. 1:**
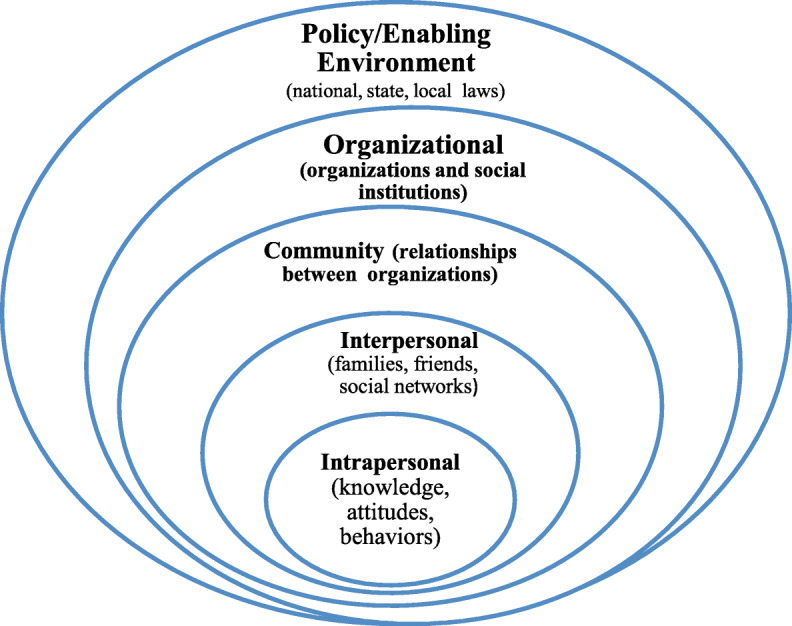
Conceptual framework of the socio-ecological model adopted from Mcleroy et al. [25]

In this study, we adopted the Socio-Ecological Model (SEM) of Mcleroy et al. [25] to understand the influences of illicit adolescent marijuana cultivation and trading in IHLM as depicted in Fig. 1. The theory was preferred due to its multi-level influences of behaviour and could extensively explore the influences of illicit adolescent marijuana cultivation and trading in IHLM. Moreover, the theory has been extensively used to attempt to understand illegal adolescent drug involvement [26, 27] and was, therefore, deemed appropriate in answering the research question. Proponents of the theory opine that a developing individual is an agent of change who can positively or negatively impact the environment in which he or she lives. Similarly, the environment could affect an individual’s development. The theory posits that five levels of environmental factors shape interactions between an individual and his surroundings; the intrapersonal, interpersonal, organisational, community and public policy levels of influence [25].

At the intrapersonal level, individuals’ age, gender, physical qualities, knowledge, skills, attitudes or intentions increase or decrease their chances of engaging or disengaging in a behaviour, independent of environmental influences. For instance, age, ignorance, poverty and idleness have been found to contribute to adolescent involvement in illicit drug activities [28, 29]. Moreover, intrapersonal level influences such as curiosity, shyness and fulfilment of one’s personal needs have contribute to illicit adolescent marijuana use [30].

Interpersonal influences, which include one’s immediate surroundings such as family, peers, neighbourhood and school or the contacts one makes at work, have direct and indirect effects on their behaviour or practice [25]. Scheier and Griffin [31] identified one’s immediate family as a significant determinate of illicit adolescent drug involvement. Similarly, peer influence has been identified as another initiator of illegal drug involvement during adolescence. According to Veliz et al. [32], adolescents are often influenced by their peers to indulge in illicit drug activities such as illegal drug usage during competitive sporting activities. Foo et al. [33] also posit that poverty often serves as a catalyst for adolescent illicit drug cultivation and trading as they are forced to earn income for their family.

Communal influences such as belief systems, illicit drug culture, geographical location and taboos, among others, could also influence adolescents to engage in illegal drug cultivation and trading. For instance, Seffrin [34] found that the composition of communities affects teenage substance use. This composition is particularly so in predominantly black South African rural communities where illicit drug activity and substance abuse abounds [35]. It has also been established that in societies where protective cultural traditions exist against illegal drug activities, adolescent drug involvement is minimised. However, where defensive cultural traditions against juvenile drug involvement are non-existent or promoted, juvenile illicit drug activities fester [36].

At the organisational level, religious bodies and civil society organisations (CSOs) operating in the community play a vital role in either influencing or reducing adolescent involvement in illicit drug activities such as marijuana cultivation and trading. In communities where religious organisations actively advocate against illicit drug use and production, the behaviour is reduced to some extent [37]. However, when religious organisations are reluctant to talk about sensitive social issues such as illicit drug involvement, such behaviours thrive [37]. According to Nasim et al. [38], the level of one’s involvement in religious activities also determines one’s level of illicit drug involvement. Thus, adolescents who regularly go to church and are involved in church activities are less involved in illicit drug activities. Aside from religious bodies, CSOs within communities also play a key role in determining illicit adolescent drug involvement. It has been established that when illicit drug prevention activities undertaking by CSO are well-coordinated, adolescent illegal involvement in drug activities such as marijuana cultivation and trading could be curtailed [39].

Lastly, policy influences, which include laws and bylaws aimed at preventing and controlling illicit drug activities, play a vital role in adolescent illicit drug involvement. Through the Children’s Act, 38 of 2005 [40], the South African constitution protects the rights of children, including adolescents, from all forms of bad behaviours. Such rights include the right of protection from being engaged and exposed to illicit marijuana cultivation, trading and usage. The South African Drugs and Drug Trafficking Act 140 of 1992 classifies marijuana as a schedule 2 drug. Hence, engaging in its cultivation and trading, irrespective of one’s age, is a criminal offence punishable by law. Offenders often pay high penalties for their involvement, including extended prison sentences [41]. Thus, contextual factors that could increase children’s vulnerability to illicit marijuana cultivation and trading in communities need to be addressed. In an attempt to address illicit drug activities and usage in the country, the Prevention and Treatment of Substance Abuse Act, No 70 of 2008 [42] was enacted to provide a comprehensive national response to combating illicit drug cultivation and trading among children. The Act requires law enforcement agents to ensure children’s safety and protection from marijuana exposure and usage through seizure, arrest of culprits and destruction of illegal marijuana plantations and products. The Act also promotes the establishment of local drug action committees at the community level to tackle the production, supply and demand of illicit drugs such as marijuana. However, ineffective law enforcement affects preventing and controlling adolescent illicit drug involvement, such as marijuana cultivation, which often stems from police corruption and complicity. In settings where the police are highly corrupt, illicit marijuana cultivation and trafficking are rife [43]. Thus, in South Africa, where the police are corrupt and complicit in illegality [44, 45], effective enforcement of policies or laws on illegal marijuana cultivation and trading could be hampered.

Moreover, research has shown that well-implemented and coordinated action plans and bylaws can curtail unhealthy illicit practices among children around the world. In Ghana, for instance, communal action plans and bylaws against child labour have yielded positive results by curtailing illegal child involvement in commercial fishing activities in some coastal communities [46]. This proves that if effectively implemented, communal action plans and bylaws could effectively address illicit adolescent practices such as illicit marijuana cultivation and trading.

## Methods

### Study setting

The study was conducted in two selected communities in IHLM of the Eastern Cape Province of South Africa. The municipality, specifically the communities, was chosen for the study because residents of the area are known to engage in illicit marijuana cultivation and trading [10]. Anecdotal evidence from the South African Police Service (SAPS) in IHLM indicated that about 256 illegal marijuana cultivation related arrests, 132 marijuana smoking-related arrests and 196 marijuana trafficking arrests were made within the municipality in 2019 alone, indicating the extent of illicit marijuana activities in the area. Hence, communities within the municipality were deemed information-rich for the study.

### Study design and participants

Focus Group Discussion (FGD) was adopted as the qualitative methodological approach to ascertain the contextual influences of adolescent marijuana cultivation and trading in IHLM of South Africa. The FGD approach was preferred because it observes the co-construction of meaning and knowledge development at the broader group level [47]. In this study, the focus group consisted of people who knew each other and shared similar marijuana cultivation and trading experiences. This was particularly useful as discussants shared their unique experiences of marijuana cultivation and trading. Also, as participants knew each other, therefore, they felt comfortable discussing sensitive issues such as illicit marijuana cultivation and trading.

Our core research team consisted of one PhD researcher (EM), two faculty members of the Department of Public Health at the Walter Sisulu University and three research assistants. The study participants comprised adolescents aged 15 to 19 years involved in illicit marijuana cultivation and trading. Adolescents who had lived in the communities for up to a year, owned a marijuana plantation or sold marijuana for commercial reasons were included in the study. However, adolescents who had travelled at the time of data collection or were seriously ill were excluded from the study. Purposive and snowball sampling techniques were used to select the communities and individual participants, respectively. In relation to the participants, initial participant(s) recruited who were identified following a lengthy period of community immersion helped in the recruitment of other participants who met the inclusion criteria.

Background checks were done on participants to ascertain the veracity of their involvement in illicit marijuana cultivation and trading. This was done by visiting the marijuana plantations of some participants and, in some cases, corroborating the participant’s account of being involved in illicit marijuana cultivation and trading by at least one verified participant. In all, thirty-three participants were recruited and interviewed for the study.

### The interview guide

A semi-structured FGD guide (Appendix I), constructed in English and translated verbatim into the IsiXhosa language, was used to conduct the interviews. The guide was developed by three authors (EM, MD and MJN). The guide was used to collect information on participants’ socio-demographic characteristics and what influenced them to engage in marijuana cultivation and trading.

The guide was piloted with five participants recruited from a community that had similar characteristics as the two communities where the main study was conducted. The pilot study was used to evaluate and revise the interview questions. This ensured the instrument’s credibility and provided insight into the best approach to use in asking questions and recruiting participants, taking into consideration the sensitivity of the study [48].

### FGD interviews

The study’s data collection proceeded after the formation of focus groups; a male focus group (MFG) and a female focus group (FFG) per community, using already established contacts in both communities. The data were collected by two trained research assistants who were conversant with the IsiXhosa language, under the guidance of the principal investigator (EM) and the second co-author (MJN), a PhD researcher and an experienced PhD supervisor, respectively, in 2 weeks after staying and interacting with community members for a month to gain their trust and establish the needed rapport. This was necessary to enable the researchers to penetrate the social networks of marijuana growers and sellers to open up to be interviewed, owing to the illegality of their business. In each focus group, alphabetical and numerical codes (e.g., MFG C1/A) were assigned to the group and participants, respectively. They were used to identify participants based on their communities and contributions during the discussion. Each interview session lasted between an hour or an hour and half. The session was recorded with an Olympus voice recorder, with the participants’ permission.

### Data analysis and saturation

Thematic content analytical techniques were employed to analyse the data, including coding for the identification of emerging themes and patterns. The data were organised by cleaning, labelling and keeping track of the different data sets from the focus group discussions. Thus, data were prepared for analysis by first transcribing and translating the interview recordings verbatim by a qualified language translator at the Eastern Cape Department of Education, Bisho, South Africa, from the IsiXhosa language to English. The research team (EM, MJN and MD) then thoroughly read through the various datasets to gain a general sense of the information and then begun to interpret their overall meaning. Colour coding of the text was then done for theme formation and categorisation. Each dataset was put into segments and codes, which were descriptive in terms of the dataset’s subject matter. Participants’ responses were coded according to their communities and focus group. Thus, male focus group responses were coded (MFG), while female focus group responses were coded (FFG). The numbers (1 and 2) assigned to each community was then placed in front of each code to indicate whether a participant was from community one or community two. The codes developed from the various datasets were then compared and arranged based on major topics, unique topics and leftovers.

Sub-themes were developed from various topics by using the most descriptive words for each category of codes. During the process, we took into account what participants meant, regardless of the terms they used, to ensure that the meanings of their expressions or words were not lost. Related topics were then grouped to reduce the number of categories and create themes. The participants’ views from all the datasets were summarised per the various themes that emerged from the analysis. Four themes, based on the constructs of the socio-ecological model [25], emerged from the data: intrapersonal influences of illicit adolescent marijuana cultivation and trading (knowledge and skills), interpersonal influences (enticement from friends who benefit financially from the marijuana business), community influences (source of livelihood, exposure to marijuana activities, orphanhood and preservation of family land) and public policy influences (lack of bylaws on marijuana activities in the municipality) were the themes derived from the data. Data saturation was deemed to have been reached when new themes did not emerge from the analysis.

### Trustworthiness

We ensured that our findings were credible, dependable, confirmable and transferable as required of qualitative research [49]. We ensured our results’ credibility by first gaining the participants’ trust by immersing ourselves in the selected communities for 1 month before data collection began. This was to ensure that potential participants trusted us enough to open up and provide credible answers, due to the study’s sensitive nature. We also ensured that our results were dependable by relying on peer debriefers and experts in qualitative research to scrutinise and critique our methods and reporting from the beginning for improvement. The study findings’ conformability was also ensured through member checking, by going back to participants to verify the transcripts and results; they agreed that what we had reported was what they said. Lastly, a chronologically detailed explanation of our methods and procedures also helped to ensure the transferability of our findings [49].

## Results

**Fig. 2:**
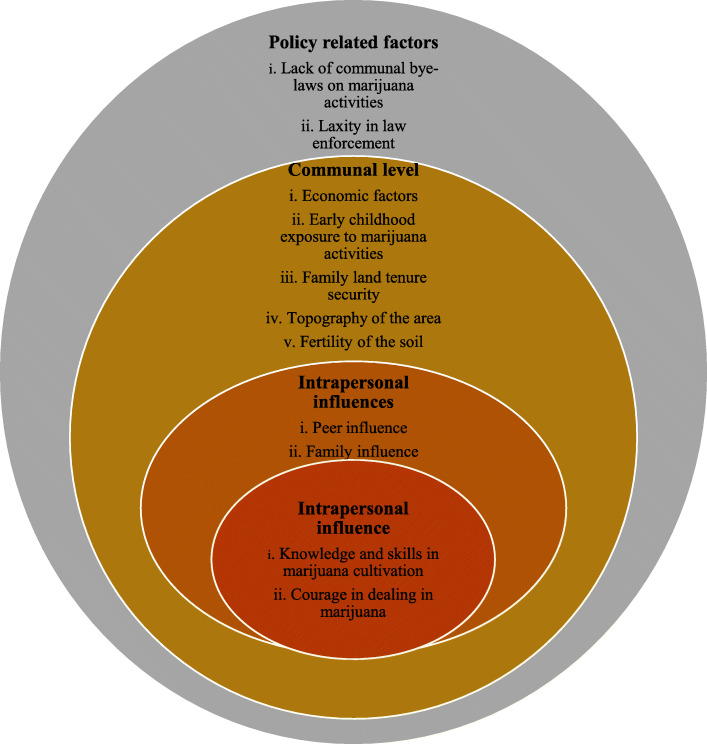
An illustration showing key findings of the study

We found that illicit adolescent marijuana cultivation and trading in the selected communities were influenced by eleven contextual influences, which are grouped under four headings based on the tenets of SEM. The results are summarised in Fig. 2.

### Socio-demographic characteristics of participants

There were thirty-three (33) participants recruited for the study. Seventeen (17) were males, and sixteen (16) were females. All the participants were between fifteen (15) to nineteen (19) years old. Most of them (23) had secondary education, while the rest had primary of education. None of the participants was formally employed. Most of them (25) were living under their parents/guardians’ care, while few (8) were living independently.

### Intrapersonal influences of illicit adolescent marijuana cultivation and trading

Analysis of the data revealed two key intrapersonal influences of adolescent marijuana cultivation and trading among discussants; knowledge and skills in marijuana cultivation and trading as well as the courage to engage in the practice.

#### Knowledge and skills in marijuana cultivation and trading

The findings revealed that one of the causes of adolescent marijuana cultivation and trading among discussants was their knowledge and skills in marijuana cultivation and trading. Discussants disclosed that their early childhood involvement in the illicit marijuana business, by either their parents, siblings or neighbours, had equipped them with both farming and marketing skills to engage in independent marijuana cultivation and trading in their adolescence. A participant explained:

*"This [marijuana cultivation] is what I grew up doing. I started working on marijuana farms when I was seven years old. It is a family business, so I know everything about the business, from planting to harvesting to transportation. I may not be able to grow mealies [maize meal] very well the same way I grow marijuana, so I have to do what I know best [to grow marijuana]" (Male, Community 1, 18 years).*

A respondent from Community 2 echoed similar sentiments by explaining how skilled he was in smuggling marijuana to cities for sale without being caught by law enforcement agents. He narrated:

*"I have been growing marijuana since I was eight years old, so I can say I am an ancestor [matured] in this business. To do something for over ten years is not easy, so what else do you expect me to do? So you see, sometimes it is not our fault, just like you are here doing your work because you have been to school, we also grow marijuana because we have been to marijuana school" (Male, Community 2, 19 years).*

#### Courage to cultivate and trade in marijuana illegally

Another intrapersonal factor that was found to influence discussants into marijuana cultivation and trading was their courage to indulge in the practice without fear. As participants have been involved in illicit marijuana activities since their childhood, they developed the courage to engage in illicit marijuana cultivation and trading for their gain without fear of being arrested. A male participant said:

*" … no, I am not afraid at all to be doing this [cultivating marijuana]. Why should I be afraid? I have been doing this since my childhood so nothing can stop me" (Male, Community 1, 17 years).*

A female participant also narrated how bold and clever she was in outwitting law enforcement agents to traffic marijuana to cities for sale. She boasted:

*"I am not afraid of the police; that is why I am in this business. Whenever I carry dagga [marijuana], I make sure that I am wearing my school uniform. In that case, even if they [police] stop the bus, they don't search me. I have been using that trick for a long time to transport dagga [marijuana] to Cape Town for my grandmother, so I am not afraid to do it, now that I have my customers" (Female, Community 2, 18 years old).*

The knowledge, skills and courage gained by discussants, in dealing with marijuana cultivation and trafficking, from their early childhood, have equipped them with the technical knowledge as adolescents to be able to cultivate and trade marijuana for their gain.

### Interpersonal influences of illicit adolescent marijuana cultivation and trading

Relevant interpersonal influences of adolescent marijuana cultivation and trading that discussants reported were peers and proximal family influences. Some discussants explained that they were lured into illicit marijuana cultivation and trading through their friends who engaged in the business. However, others said they did so because their families were into the business.

#### Peer influence

Concerning peer influence, it was identified that peers of participants who benefitted financially from their engagement in marijuana activities were seen to be living affluent lifestyles than those who were not. Consequently, participants were enticed by the ostentatious lifestyles of their peers and followed suit, as explained by a sixteen-year-old participant:

*"At school, those boys who sold marijuana wore quality snickers and shoes. They always had everything they wanted while I was often hungry and looked dirty. So, I also wanted to look good like them [marijuana growers], so I started cultivating my dagga after school and on weekends in the valley" (Male, Community 2, 16 years).*

A female respondent from Community One also retorted:

*"I used to envy my friend who used to help her mother sell marijuana. She was always looking neat at school, buying sweets for us, so I got jealous and decided to get involved in the marijuana business. My family had no farm, so I started by helping my neighbours on their farms. That is how I learnt how to grow and sell marijuana" (Female, Community1, 18 years).*

#### Family involvement in marijuana activities

Some participants indicated that they come from families whose main economic activity is illicit marijuana cultivation and trading. They see it as the only economic venture they can undertake and, therefore, they have no remorse in doing so as they are involved in raising the needed family income. A female participant from Community One explained:

*"It [marijuana cultivation and trading] is a family business, so I am just continuing with the tradition [of illicit marijuana cultivation]. As you can see, there are no jobs here [in the community], so this is what our parents used to raise us, and now that I am old, what stops me from having my own plantation?" (Female, Community 1, 17 years).*

Another participant narrated how he got involved in the marijuana business through his elder brother. He said:

*"It was my elder brother who introduced me to this [marijuana cultivation and trading]. He was the only one who used to cultivate dagga [marijuana] in the family, although my parents opposed it. But as a stubborn boy, I used to follow him to his plantation and ended up cultivating it [marijuana] for my personal gain" (Male participant, Community 2, 18 years).*

Hence, having peers who engaged in illicit dagga cultivation or coming from households or families where illicit dagga practices abounded influenced discussants into illicit dagga cultivation and trading.

### Community influences of illicit adolescent marijuana cultivation and trading

#### Economic reasons

One key communal influence of illicit adolescent marijuana cultivation and trading was economic reasons. Discussants stated that their only source of income was cultivating and trading marijuana as impoverished and economically disadvantaged communities. Illicit marijuana cultivation and trading are the main economic activity of the area; hence, there is a ready market for marijuana, making it the ideal economic commodity to trade. A female participant explained that:

*"It [marijuana] is the only thing that brings us money here. As you can see, there are no jobs here. The government has disappointed us, so if you are here and don't want to grow or sell dagga [marijuana], you have to go to the mines, and these days, it is not easy to get a job at the mines, especially for women. That is why we all have decided to grow dagga [marijuana] to survive" (Female, Community 1, 19 years).*

A male discussant from Community Two shared similar sentiments. He narrates how their community is known for its quality marijuana countrywide, hence, it serves as their main source of income. He narrated:

*"This [marijuana cultivation] is what this place [municipality] is known for because it is our only source of livelihood. We don't have any white-collar jobs here, except the few teachers and police in the town, but they are all not from here. And as a human being, you need to survive. Thankfully, the quality of our dagga [marijuana] is appreciated countrywide, so we make a lot of money from it" (Male, Community 2, 18 years).*

Thus, the lack of economic opportunities in the area has made illicit marijuana cultivation and trading the only profitable alternative for community members, and therefore, has enticed discussants to engage in it to earn a living.

#### Early childhood exposure to marijuana cultivation in the communities

Early childhood exposure to marijuana cultivation in the two communities was another major community influence with regard to illicit adolescent marijuana cultivation and trading. Marijuana cultivation in both communities was a cultural norm as well as an economic one. Discussants grew up seeing marijuana cultivation all around them in their communities. They, therefore, automatically participated in it. A female participant from Community One explained that:

*"We [children] grew up and saw almost everyone [community members] having a marijuana field or selling it [marijuana]. We saw it [marijuana] from our parents and neighbours, so we also copied and cultivated it. I don't have my own [marijuana] farm yet, but I help my mother on her [marijuana] farm" (Female, Community 1, 16 years).*

To a male participant from Community Two, marijuana cultivation and trading is automatically learnt as the plant has been cultivated over generations in their community, making it common and popular in the area. He explained that:

*"No one teaches you how to cultivate marijuana; it is automatically learnt because it can be found everywhere in this community. We [community members] have been cultivating this plant [marijuana] for a long time so it like our inheritance, you cannot grow up here [in the municipality] and say you do not know how to cultivate marijuana" (Male, Community 2, 16 years).*

#### Marijuana cultivation as a means of protecting and preserving family land from encroachers

Adolescents sometimes have to cultivate marijuana on their family lands to prevent such lands from being encroached on by other illegal marijuana growers. Marijuana-related land disputes are not usually reported for amicable settlements, hence, one’s land was likely to be taken over if left to lie fallow, that is to say, without growing marijuana on it. A female participant from Community Two recounted her experience:

*"Sometimes you have to cultivate your family land if you are the older person left at home, else, by the time you realise, someone would have taken over your [family] land. You know this is not a thing [marijuana-related land dispute] you can report to anyone, not even the chief because when it gets to the police or court, what are you going to say?" (Female, Community 2, 18 years).*

A male participant from the same community shared similar sentiments:

*"With some of us, our parents are dead, and we are now the heads of our families so if we don't grow marijuana on the land that was left to us, some other families will take over and that will be our end. Once the land is lying fallow for a long time, outsiders will just claim it [by growing marijuana on it], so we have to protect it" (Male, Community 2, 17 years).*

#### The topography of the area

It was also established that adolescents indulged in marijuana cultivation and trading due to the undulating topography of the area. Discussants explained that the area’s hilly nature provides them with enough cover to cultivate marijuana at the blind side of law enforcement agents as agents often find it difficult to enter the deep valleys where marijuana is cultivated. A participant recounted:

*"I will say this place [the locality] is very good for dagga [marijuana] business because the place is mountainous with a lot of valleys so if you manage to have a farm in one of the valleys, the police will not see it. Even if they are aware, they [police] sometimes find it very difficult to descend and climb the hills, so they give up on you" (Male, Community 1, 17 years).*

Another discussant further explained:

*"This place [the locality] is too hilly and dangerous for a policeman to risk his life and come after us down there [in the valleys] to arrest us, so they often don't bother. It is only the helicopters which can do that job [destroy marijuana plantations in the valley] and even those, sometimes they don't succeed" (Male, Community 1, 19 years).*

#### The fertility of the land

The fertility of the areas was also mentioned as a contextual factor influencing adolescent marijuana cultivation and trading. Discussants believed that although the soil was fertile for other crops, it was more fertile for marijuana. They, therefore, had no choice but to grow marijuana to benefit from the soil. A discussant narrated:

*"I will say it is because of the soil. It is so fertile that once you grow dagga [marijuana], you don't need to apply fertiliser. It can grow on its own. But if you grow other crops like potato or maize, you will have to apply fertiliser, else you will just waste your energy" (Female, Community 1, 18 years).*

Another participant shared his experience by explaining why he switched from maize farming to marijuana cultivation. He narrated:

*"I once cultivated maize by trying to be a good guy. However, the rains did not fall as they should, so I run at a loss. In the end, I had to resort to borrowing money from my friends who grow marijuana since they make a lot of money. So you see, even with little water in the soil, marijuana will still grow" (Male, Community 2, 16 years).*

Lack of alternative economic activities in the communities has made families resort to illicit marijuana cultivation and trading, making the plant readily available in the area. As a result, lands have become a precious commodity that one has to preserve by consistently growing marijuana on it to ward off encroachers. Furthermore, the topography of the area made it ideal for illicit marijuana cultivation and trading as the undulating nature of the area makes it inaccessible to law enforcement agencies to destroy and arrest its growers. Also, the fertility of the land favours marijuana compared to crops such as maize. These cumulative communal factors influenced discussants into illicit marijuana cultivation and trading.

### Policy-related influences of illicit adolescent marijuana cultivation and trading

Two key policy influences were identified to be responsible for illicit adolescent marijuana cultivation and trading. The lack of communal bylaws to control illegal marijuana activities as well as negligence on the part of law enforcement agents have encouraged adolescent marijuana cultivation and trading to thrive.

#### Lack of communal bylaws in controlling illegal marijuana activities

The culture of marijuana cultivation and trading in the two communities seemed to be endorsed by community leaders as there were no bylaws or enforcement of state laws to curb the act. As such, discussants took advantage of the opportunity to grow and trade in dagga. A discussant from Community Two explained why they only feared the police and not community leaders:

*"We are only afraid of the police who sometimes come here with helicopters to spray our fields, not the local authorities because there are no laws here that stop anyone from cultivating marijuana" (Male, Community 2, 17 years).*

Another participant from Community Two explained how local authorities had conceded to the marijuana business, rendering them powerless to enact and enforce bylaws on marijuana cultivation and trading.

*"There are no laws here that ban anyone from growing marijuana. Every family has a marijuana field. Even if the chief doesn't have one, some of his family members may have, so who is he [the chief] going to stop from cultivating marijuana?" (Male, Community 2, 18 years).*

#### Laxity in law enforcement on illicit marijuana activities

Another policy influence of adolescent marijuana cultivation and trading was laxness in law enforcement in the communities. Although there is police presence in the area, participants were confident of growing marijuana without being apprehended. They believed that their farms would not be destroyed or would they be arrested as some police officers’ benefit from the marijuana business, and therefore, would not arrest them. One participant explained:

*"My brother, some police officers here were even educated with proceeds from marijuana, so how can they come and destroy our farms or arrest us? They also benefit from it [marijuana cultivation and trading], so they cannot do anything about it [marijuana cultivation]" (Male participant, Community 2, 19 years old).*

Another participant explained how they continue to operate amid police and other law enforcement agents. He narrated:

*"Sometimes they come to spray the farms, but we know this doesn't happen often, so we are not worried. Besides, some of the police are our friends, so when they [law enforcement agents] are about to raid our homes or block the roads, we are tipped off to hide our goods. Some of them are just pretending to be serious on us, but the truth is that they help us a lot" (Male participant, Community 1, 18 years old).*

## Discussion

This paper explored the contextual influences of illicit adolescent marijuana cultivation and trading in two communities in IHLM, South Africa. Eleven contextual influences, grouped under four levels of influence; intrapersonal, interpersonal, communal and public policy influences, were found to be associated with illicit adolescent marijuana cultivation and trading.

Intrapersonal factors such as technical knowledge on marijuana cultivation through their early childhood involvement in illicit marijuana activities equipped adolescents to venture into marijuana cultivation. By the time children reached adolescence, they had the requisite knowledge, skills and courage to cultivate, traffic or sell marijuana. Buttressing this assertion, Xu et al. [50] posit that there is a link between good knowledge and subsequent behaviour practice. Thus, when people are knowledgeable and skilful about an activity, they usually attain self-efficacy and become confident enough to indulge in it. Hence, adolescents’ involvement in marijuana cultivation and trading in their formative years empower them with the prerequisite skills and confidence to cultivate and sell marijuana. Thus, the experiences children gain from exposure to illicit drug activities such as marijuana cultivation and trading often lead to future involvement in such a trade as these experiences translate into knowledge on how such activities are handled [51]. Therefore, the exposure and involvement of children in marijuana related activities in our study communities need to be urgently curtailed to prevent future involvement in illicit marijuana activities.

Another salient intrapersonal factor was participants’ courage to engage in illicit marijuana cultivation and trading. Most of the participants were involved in illicit marijuana cultivation and trading in childhood, therefore, they were empowered by the time they reached adolescence to cultivate or sell marijuana on their own. Studies have shown that when individuals develop greater courage, they can engage in behaviours that they were formerly afraid of [52]. In further explaining the role courage plays in influencing task involvement, Chockalingam and Norton [53] opine that when courage is high, the anxiety associated with engaging in previously fearful actions or behaviours is reduced, thereby enabling one to do such a thing. Therefore, as discussants consistently cultivated and traded marijuana, they developed high courage and became bold to engage in illicit marijuana cultivation and trading. The bravery of discussants to engage in illicit marijuana cultivation and trading could also stem from the competence they have gained over the years trading in marijuana [54]. Therefore, children and adolescents in the selected communities need a constant reminder of the dangers of illicit practices to prevent them from engaging in such practices.

Concerning interpersonal influences, peer influence was a strong motivator of adolescent marijuana cultivation and trading in the two communities. Adolescents from households that cultivate marijuana portrayed an affluent lifestyle at school, which enticed those who were not involved in it to follow suit since they also wanted to live comfortable lifestyles as their peers. In explaining the influence peers have on adolescents’ involvement in behaviour, Loke and Mak [55] mention that peer influence is a highly predictive factor of whether or not adolescents will engage in behaviours such as marijuana cultivation. This is so because adolescents tend to emulate the actions of their peers. As such, teenagers who see their peers benefitting financially from illicit marijuana cultivation and trading will be compelled to do same. Moriarty and Higgins [56] further explain that behavioural influence is strong among teenagers who have stable friendship networks. Hence, adolescents from a rural community where friendship networks are more stable and stronger [57] are likely to engage in illegal marijuana cultivation if their peers were involved in such a practice. Therefore, promotion of positive peer influence in communities should be of paramount interest if adolescent marijuana cultivation and trading are to be addressed.

Also, participants’ families’ involvement in illegal marijuana cultivation was found to have exposed and initiated them into the act. In most families, illegal marijuana cultivation is their only source of income due to the lack of employment opportunities. Hence, adolescents appear to have no choice but to engage in illegal marijuana cultivation and trading to help raise much-needed family income. This involvement subsequently has taught and influenced them to get involved in illicit marijuana cultivation and trading either for their own or family’s benefit. Bharadwaj [58] explains that although poverty cannot be the sole underlying cause of involvement in illegal activities such as marijuana cultivation, it is the most significant factor, especially when people run out of alternative sources of living. This was the case in the two communities in IHLM, South Africa. While a few families receive remittances from their relatives who work in the various mines across the country, as well as social grants from the government, the money was not enough to cater for the ever-increasing needs of households [59]. Adolescents were, therefore, pushed into illegal marijuana cultivation by their families to augment their meagre family income and were trapped in the trade.

Concerning community influences, the economic reason was found to have motivated discussants to engage in illicit marijuana cultivation and trading. Economic dependence on illicit drug production and sale in impoverished settings has been documented in countries such as Afghanistan [60] and has been long documented in IHLM. Most Black South African communities are so economically disadvantaged that engaging in criminal activities for survival is common as the pace of economic transformation in such communities has been very slow since independence [61, 62]. This often pushes families into illegal marijuana cultivation and trading in some communities for survival and invariably, pushes adolescents into the trade. Hence, to address adolescent marijuana cultivation and trading in our study communities, economic opportunities need to be provided to decrease their interest in the illicit marijuana business.

Moreover, due to the widespread cultivation and trading of marijuana in the two communities, it is widely available in the area. This widespread availability of marijuana exposes adolescents to marijuana activities, such as cultivation and trading daily, thereby, influencing them to engage in such practices over time. Adolescents see the marijuana plant being cultivated and traded in their immediate environment, and thus, know its economic importance. Their immediate environments and neighbourhoods were, therefore, infested with the culture of marijuana cultivation and trading, which according to Bronfenbrenner [63], has a profound influence on what a child would become in the future. If a child’s environment is a toxic one infested with a social vice, the child is more likely to grow and engage in the practice. Hence, as adolescents grow up in communities where marijuana is grown, they became accustomed to the practice and indulged in it. Mcleroy et al. [25] explained in their SEM that a child is both the product and the producer of his/her environment. In other words, the environment influences a child’s development while the child, in turn, influences his environment by creating the type of environment in which s/he prefers to live. Adolescents, however, might not influence change in their environment as they are too young to decide for themselves, let alone to influence an entire community. Thus, as participants lacked the influence to change their environment, they became products of it by partaking in their communal tradition of illegal marijuana cultivation and trading.

Another community level influence of illicit adolescent marijuana cultivation and trading was protecting family land from potential encroachers. In some child-headed households, teenagers sometimes ventured into marijuana cultivation as a means of protecting their family lands from encroachers who might capture lands lying fallow for illegal marijuana cultivation. Hence, no matter how young an adolescent was, he or she had to lay claim to their family land to prevent strangers from taking over. This was compounded by the lack of access to land for agrarian purposes in post-apartheid South Africa. As most people lost their lands in the historical past, and with a growing call for land reforms in the country [64], individuals go to great lengths to protect their lands from being taken over. Thus, everyone, including adolescents, will do whatever it takes to own or prevent their lands from being taken over, even if it means illegally growing marijuana on it. As such, adolescents engage in marijuana cultivation in the two communities as a way of protecting their inheritance. Moreover, once an individual has access to land, the tendency of using it for economic activities, including illegal marijuana production, is very high [13]. The implication is that a proper land tenure system for individuals and families in rural communities could protect and prevent illegal takeovers of lands, especially for child-headed households, and thus, minimise adolescent involvement in illegal marijuana cultivation and trading.

Furthermore, the topography of the area was found to be another factor that influences illicit adolescent marijuana activities. Discussants explained that the area’s undulating nature enabled them to cultivate marijuana in areas inaccessible to law enforcement agents and, thus, were at little risk of being arrested and their plantations being detected and destroyed. Cultivation and production of illicit drugs in remote public lands is a common practice among drug traffickers. Such environments make it difficult for the identification and destruction of marijuana plantations. In Mexico, for instance, topography was found to be one of the environmental factors associated with illicit marijuana cultivation as drug traffickers often cultivated marijuana in inaccessible wetlands [65]. While little can be done to change the topography of the area, provision of alternative livelihood schemes coupled with consistent education on the dangers of illicit marijuana cultivation and trading could sway community members from engaging in illicit marijuana cultivation and trading [66].

Furthermore, discussants posited that soil fertility was a contributing factor for their involvement in illicit marijuana cultivation and trading. They disclosed that the land mostly suited marijuana than other food and cash crops such as maize, and thus, offered them an easy choice between marijuana and other crops. They further explained that the marijuana plant could withstand drought conditions unlike other crops and therefore, they are able to grow it even in the dry season. Discussants’ explanation of marijuana’s ability to thrive in poor soils, thus, influences their decision to engage in its cultivation. However, this assertion is not supported in the literature. On the contrary, marijuana requires highly fertile soil and large volumes of water to grow well [67]. The high nutritional demands of marijuana have led to traffickers clearing large tracts of fertile forest lands for its cultivation in Columbia [68]. This implies that discussants might be using fertile arable lands for food crop production for marijuana purposes with the misconception that the soil is less fertile for food and cash crops. As a result, education on the nutritional demands of various crops needs to be intensified in the communities to enable them to make informed choices on crops that will best suit the soil other than marijuana.

About policy influences, the lack of communal bylaws that regulate illegal marijuana activities by prohibiting adolescents’ involvement in marijuana cultivation and trading in the two communities and the laxity of law enforcement agents in enforcing existing laws have encouraged adolescents to engage in illicit marijuana cultivation and trading. Although marijuana has been legalised for private use, its cultivation and trading for commercial gains remain illegal [69]. However, despite the long-standing illegality of marijuana cultivation and trading, the practice has been on-going in IHLM, with community leaders not frowning on the act. There are no communal bylaws or restrictions that prohibit or at least prevent adolescents from engaging in the act.

Lastly, laxity in enforcing laws on illicit marijuana cultivation and trading in the two communities has emboldened adolescents to engage in it. Although sporadic destructions of marijuana plantations are carried out by law enforcement agents [70], it does not deter them from growing marijuana illegally. Also, participants made no reference to arrests in their communities about illegal marijuana cultivation. Marijuana-related arrests often made by police were in relation to its transportation for sale. Hence, laxity in law enforcement about marijuana cultivation seem to encourage adolescents in the two communities to engage in illegal marijuana cultivation. Law enforcement agents have long been identified as key role players in the fight against the illicit drug trade [71, 72]. Thus, should the police renege on their mandate or become complicit, the illicit drug trade fester. According to Goga [73], some people involved in the illicit drug trade in South Africa wield political or economic power, making it difficult for law enforcement agents to strictly enforce the law. Thus, such individuals sometimes influence law enforcement officers to turn a blind eye to their dealings or are too powerful to be arrested. In some instances, law enforcement agents become complicit in the illegal marijuana business [44, 45], compromising their integrity and stance to genuinely fight crime. In such situations, adolescents are bold enough to be involved in illegal marijuana dealings. Hence, it could be argued that the lack of proper policy or law implementation in addressing illicit marijuana activities is partly responsible for adolescent involvement in marijuana cultivation and trading in the two communities and need to be addressed.

### Policy implications

Our findings reveal a policy implementation lapse regarding demand and supply reduction of marijuana in the IHLM. The Prevention and Treatment of Substance Abuse Act, No 70 of 2008 [42] aims to provide a comprehensive national response for combating substance abuse through several measures such as demand reduction and early prevention through a multi-sectorial approach. The Act empowers and demand law enforcement agents to seize and destroy marijuana and also arrest culprits. The Act also promotes and empowers the establishment of local drug action committees at the community level to tackle the production, supply and demand of illicit drugs. However, it is evident from our findings that law enforcement on supply reduction for marijuana is minimal in IHLM. This could be as a result of the complicity of bylaw enforcement agents in illicit drug networks for financial gain on the African continent in general [74], considering the high level of corruption reported among the SAPS [75, 76].

Moreover, drug policy development and implementation in South Africa, over the years, has been under the auspices of various government departments and the Central Drug Authority (CDA), with the National Drug Master Plan (NDMP) being the single most significant document in addressing substance abuse related issues in the country. However, certain weaknesses have been highlighted in the NDMP that make its effectiveness in addressing substance abuse challenges in the country through policy implementation ineffective. For instance, the NDMP does not translate generic policy statements into clear recommendations for action, a task that has been shifted to the provincial level, resulting in major differences in drug policy between provinces, both in terms of the stage of policy development and implementation [77]. Furthermore, lack of leadership on drug-related issues, both at national and provincial levels, is another key challenge facing the NDMP, leading to no person being responsible for driving the implementation of policies or accountable for the successes and failures of policies at the provincial level [77]. This could be the case for law enforcement agents in IHLM in their effort to combat the supply of marijuana, as there could be the lack of a focal person who is responsible for driving the implementation of policies at the local level, taking into consideration the dangers associated with fighting crime in South Africa [78].

Furthermore, policies or programmes directed at reducing consumer demand for psychoactive drugs are either educative, treatment or rehabilitating in nature as opposed to law enforcement strategies, per the NDMP [1]. Therefore, this approach encourages illicit drug users to solicit for drugs, including marijuana, thwarting supply reduction efforts as there is a market for the drug. Collaborative efforts between law enforcement agents and communities, per the NDMP, are also non-existent in IHLM as deduced from our findings. This lack of community-led initiatives in preventing adolescent and general community involvement in illicit marijuana cultivation and trading, coupled with laxity in law enforcement on illicit marijuana cultivation and trading, encourages the practice to fester. Thus, in this context, The Prevention and Treatment of Substance Abuse Act, No 70 of 2008, through the NDMP, has failed to protect children’s rights in our study communities. Hence, the identified lapses highlighted in the NDMP need to be urgently addressed to effectively coordinate demand and supply reduction strategies for marijuana in the communities and the municipality.

## Conclusion

Four ecological influences of adolescent illicit marijuana cultivation and trading in the two communities of IHLM were found, ranging from intrapersonal influences, interpersonal influences, community influences to lapses in law enforcement. It is, therefore, recommended that substance abuse prevention policies and programmes focus on discouraging child involvement in illicit marijuana activities in IHLM across the four tenets of the SEM to curtail adolescent involvement in marijuana cultivation and trading. There is also the need to incorporate the law enforcement approach into the demand reduction strategies of the NDMP, which employs only an educative approach in its current form. Working agreements between municipal authorities, law enforcement agents and social service professionals also need to be strengthened in order to coordinate demand reduction strategies for marijuana in the communities to protect the rights of children as enshrined in the Children’s Act, 38 of 2005.

## Data Availability

The datasets used and analysed during the current study was available from the corresponding author on reasonable request.

## Abbreviations

CDA: Central Drug Authority
CSOs: Civil Society Organisations
EC: Eastern Cape
FFG: Female Focus Group
FGD: Focus Group Discussion
IHLM: Ingquza Hill Local Municipality
KZN: Kwa-Zulu Natal
MFG: Male Focus Group
NDMP: National Drug Master Plan
SAPS: South African Police Service
SEM: Socio-Ecological Model.
